# Altered mRNA Levels of Stress-Related Peptides in Mouse Hippocampus and Caudate-Putamen in Withdrawal after Long-Term Intermittent Exposure to Tobacco Smoke or Electronic Cigarette Vapour

**DOI:** 10.3390/ijms22020599

**Published:** 2021-01-09

**Authors:** Lucia Carboni, Luisa Ponzoni, Daniela Braida, Mariaelvina Sala, Cecilia Gotti, Michele Zoli

**Affiliations:** 1 Department of Pharmacy and Biotechnology, Alma Mater Studiorum—Università di Bologna, 40126 Bologna, Italy; 2 Department of Medical Biotechnology and Translational Medicine, Università degli Studi di Milano, 20133 Milan, Italy; luisa.ponzoni@guest.unimi.it (L.P.); daniela.braida@guest.unimi.it (D.B.); mariaelvina.sala@unimi.it (M.S.); 3 Institute of Neuroscience, CNR, 20162 Milan, Italy; cecilia.gotti@in.cnr.it; 4 Center for Neuroscience and Neurotechnology (CfNN), Department of Biomedical, Metabolic and Neural Sciences, University of Modena and Reggio Emilia, 41124 Modena, Italy; michele.zoli@unimore.it

**Keywords:** e-cigarette, nicotine addiction, addiction models, nociceptin, opioid peptides, opioid receptors, orexin receptors, Crf, Crf receptors, Bdnf

## Abstract

Nicotine addiction is a severe public health problem. The aim of this study was to investigate the alterations in key neurotransmissions after 60 days of withdrawal from seven weeks of intermittent cigarette smoke, e-cigarette vapours, or an e-cigarette vehicle. In the nicotine withdrawal groups, increased depressive and anxiety/obsessive–compulsive-like behaviours were demonstrated in the tail suspension, sucrose preference and marble burying tests. Cognitive impairments were detected in the spatial object recognition test. A significant increase in Corticotropin-releasing factor (Crf) and Crf1 mRNA levels was observed, specifically after cigarette withdrawal in the caudate-putamen nucleus (CPu). The nociceptin precursor levels were reduced by cigarette (80%) and e-cigarette (50%) withdrawal in the CPu. The delta opioid receptor showed a significant reduction in the hippocampus driven by the exposure to an e-cigarette solubilisation vehicle, while the mRNA levels doubled in the CPu of mice that had been exposed to e-cigarettes. Withdrawal after exposure to e-cigarette vapour induced a 35% Bdnf mRNA decrease in the hippocampus, whereas Bdnf was augmented by 118% by cigarette withdrawal in the CPu. This study shows that long-term withdrawal-induced affective and cognitive symptoms associated to lasting molecular alterations in peptidergic signalling may determine the impaired neuroplasticity in the hippocampal and striatal circuitry.

## 1. Introduction

Tobacco smoking is extremely widespread, with 1.07 billion smokers in the world as of 2019 [1], and it is recognised as a main cause of preventable deaths and diseases. Tobacco-related deaths amount to more than 8 million people a year, as the result of both direct tobacco use and indirectly, due to non-smokers being exposed to second-hand smoke [2,3]. The adverse health consequences of smoking impinge on most organs, and also including fetal development, with the largest impact being exerted on the onset of smoking-related cancers, cardiovascular and metabolic diseases, and pulmonary diseases [4].

Smoking is maintained notwithstanding the awareness of the health costs because nicotine, the main active ingredient of cigarettes (cig), is highly addictive [5,6]. Nicotine is a natural alkaloid that exerts its pharmacological effects by binding to and activating the nicotinic receptors of the neurotransmitter acetylcholine in brain. Nicotinic receptors are pentameric ion channels made up of five subunits that allow a cation influx into the neuron in response to agonist binding, thus regulating neuronal activity [7,8]. Nicotinic receptors which are especially relevant for addictive properties are located in the mesocorticolimbic pathway, in which they modulate dopaminergic signalling from the mesencephalic dopaminergic regions to the striatal and cortical areas, as well as glutamatergic and GABA-ergic signalling [9]. The mesocorticolimbic dopaminergic system controls reward and reinforcement processing, motivation, and goal-directed behaviours. Nicotine increases the dopamine levels in the mesocorticolimbic system, influencing synaptic plasticity in this circuit [5,9].

The chronic activation of nicotinic receptors promotes the development of neuroadaptations aimed at counterbalancing the excess dopaminergic activation as a homeostatic response. A first step in this process is the observed up-regulation of nicotinic receptors, which modulates the activity of a plethora of other neurotransmitters and neuromodulators, thus influencing the mesocorticolimbic pathway as well as a widespread network of other circuits [5,7,8]. The neuroadaptations resulting from chronic nicotine exposure encompass alterations of opioidergic signalling, including dynorphin-ergic and enkephalin-ergic circuits [10,11], as well as nociceptin [12,13], corticotropin-releasing factor (Crf) [14,15], brain-derived neurotrophic factor (Bdnf) [16], and orexin/hypocretin signalling [17,18].

The development of counter-adaptations to nicotine-triggered alterations to the brain circuits brings about a dysregulation of responses when nicotine assumption is abruptly interrupted, which manifests as withdrawal syndrome. Nicotine withdrawal syndrome includes affective symptoms such as anger/irritability, anxiety, depression, and nicotine craving; cognitive symptoms like difficulty concentrating and decreased alertness; and somatic symptoms like bradycardia, constipation, weight gain, and insomnia [19,20]. The duration of the syndrome appears to be 2–4 weeks, although subgroups of smokers follow different time courses, including no decline over time [19]. The emergence of a withdrawal syndrome with negative affective symptoms acts as a negative reinforcement mechanism, thus contributing to the maintenance of smoking behaviour [5,20]. As a aid to quit smoking while reducing withdrawal symptoms [21], e-cigarettes (e-cig) have become most popular in recent years. However, still imprecisely-known health challenges are posed by e-cigarettes, which in turn can induce dependence and withdrawal symptoms [22,23].

Animal models provide a valuable contribution to the comprehension of the molecular underpinning of nicotine dependence and withdrawal, and are also aimed at the identification of new targets for pharmacological intervention [5,9,24]. The available evidence shows that nicotine dependence can be induced in rodents with repeated nicotine administrations via different routes. Withdrawal syndrome can also be modelled, either by discontinuing nicotine administration or by administrating nicotinic receptor antagonists. Withdrawal symptoms in rodents include cognitive, affective, and somatic symptoms [24,25]. The withdrawal syndrome in nicotine-containing smoke rodent models has been characterised for up to 30 days after the nicotine delivery interruption [26,27].

In an effort to extend the time-course of the withdrawal syndrome, we recently carried out longer-term studies in mice that had been exposed to e-cigarette vapour or tobacco cigarette smoke for seven weeks. We examined both the affective and cognitive symptoms. As regards the affective component, we tested anxiety-like-behaviour using the elevated plus maze task; compulsive-like behaviour, using the marble burying test; depressive-like behaviour, using the tail suspension test; and the anhedonic level, using the sucrose preference test. Cognitive deficits were evaluated with the spatial object recognition task. We discovered that spatial memory deficits, increased anxiety, and compulsive-like behaviour were induced at early time-points and persisted for up to 90 days. Remarkably, attention-related and depressive-like behaviours emerged later, and were equally long-term persistent [28].

The aim of the present study was to investigate the molecular correlates underlying the neurobiological mechanisms of the behavioural alterations observed at late time-points of withdrawal. For this objective, we examined the gene expression of the neuropeptide systems involved in the neuroadaptations which develop in response to chronic nicotine administration [10,11,12,13,14,15,16,17,18], as their involvement in long-term withdrawal from cigarette smoke and e-cigarette vaping is unknown. We investigated a 60-day withdrawal because our previous studies showed that the behavioural and molecular impairments that mimic the main aspects of human withdrawal syndrome were still visible at this long-term time-point [28].

## 2. Results and Discussion

### 2.1. Behaviour

Mice were exposed to intermittent cigarette smoke, e-cigarette vapours or an e-cigarette vehicle for three 30-min sessions/day for seven weeks. The mice in the control group were exposed to air in the same cages, and with the same schedule. Sixty days after the last treatment or air exposure, the animals underwent behavioural tests that were meant to probe the affective and cognitive correlates of withdrawal (Supplementary Figure S1).

**Figure 1:**
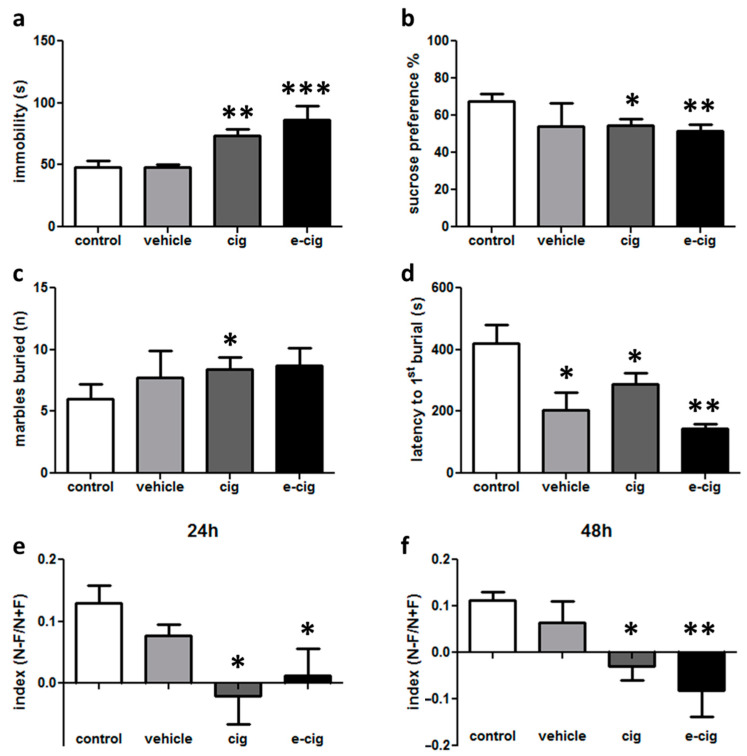
Depressive-like behaviour evaluated in the cigarette (cig), e-cigarette (e-cig) and vehicle mice compared to the control group at 60 days of withdrawal: (**a**) immobility time evaluated for 6 min in the tail suspension test; (**b**) sucrose preference measured in terms of the percentage of the consumed sucrose solution to the total volume of liquid consumed. The evaluation of anxiety-like behaviour in the marble burying task as the total number of buried marbles (**c**) and latency to the first burial (**d**) evaluated within 15 min. Cognitive impairments evaluated in the spatial object recognition test as the discrimination index at a 24 h (**e**) or 48 h delay (**f**). *n* = 4–32 for each group. *** *p <* 0.001; ** *p <* 0.01, * *p <* 0.05 in the Planned Comparison vs. control.

The onset of affective impairments was evaluated in different tests aimed at assessing distinct dimensions. Depressive-like behaviours were evaluated in the tail suspension and sucrose preference tests. The tail suspension test measures the adoption of a passive response in a stress situation as the amount of time spent by the mouse in an immobile posture. This parameter is sensitive to being reduced by the administration of anti-depressant medications. After the 60-day withdrawal from cigarette smoke or e-cigarette vaping, the immobility duration was significantly longer in both the cigarette and e-cigarette groups with respect to the controls, with no difference in the vehicle group (Figure 1a).

The sucrose preference test aims to evaluate anhedonia, one of the core symptoms of major depression. This test is based on the natural preference of mice for sweet tastes, assuming that this preference is proportionate to the pleasure that the animal experiences in consuming sweet solutions. Thus, a reduced preference is considered to reflect a reduced capacity to experience pleasure. In our experimental design, significantly decreased sucrose preference was detected in the cigarette and e-cigarette groups compared to the controls (Figure 1b). No difference was revealed in the vehicle-treated mice (Figure 1b).

In the marble burying test, which probes anxiety- or obsessive–compulsive-related behaviours, the spontaneous behaviour of burying objects in the cage bedding is measured, because this activity is intensified in conditions that are perceived as threatening. After the 60-day withdrawal, we observed that the number of buried marbles was significantly higher in the cigarette group compared to the controls (Figure 1c). Moreover, the latency for starting the burying behaviour was significantly shorter in all of the treated groups with respect to the control group (Figure 1d).

Overall, these results show that alterations in depressive- and anxiety/compulsive-like behaviours can be detected 60 days after the cessation of exposure to cigarette smoke or e-cigarette vapours. In addition, although statistical significance was not reached, these findings provide hints that e-cigarette vehicle may influence behavioural responses, as observed in the sucrose preference and marble burying tests (Figure 1b,c). Therefore, future studies should be dedicated to the expansion of the comprehension of the effects of inhaled substances on anxiety-like behaviours. Moreover, these data suggest that e-cigarette models should always include a comparison with an e-cigarette vehicle group in addition to the air-inhaling controls.

In addition to the measurement of the affective symptoms, cognitive impairments were also measured. The spatial object recognition test relies on rodents’ innate preference for novelty: mice that remember the locations in which two objects were presented during the training phase will preferentially explore the displaced object in the test session. Therefore, a reduced discrimination index indicates that the animal is affected by a spatial memory deficit. In our experimental setting, we discovered that both the mouse group exposed to cigarette smoke and that exposed to e-cigarette vapours showed a reduced discrimination index at 24 h with respect to the control group (Figure 1e). Likewise, the impairment was still severe at a 48 h delay (Figure 1f). No effect was detected for the vehicle group (Figure 1e,f). No difference between the groups was found in the total exploration time (not shown).

These results show that mice were still affected by memory deficits at 60 days after the cessation of nicotine exposure, thus modelling the findings that cognitive impairments can be observed in human abstinent smokers for extended periods of time [19,20].

### 2.2. Crf System

**Figure 2:**
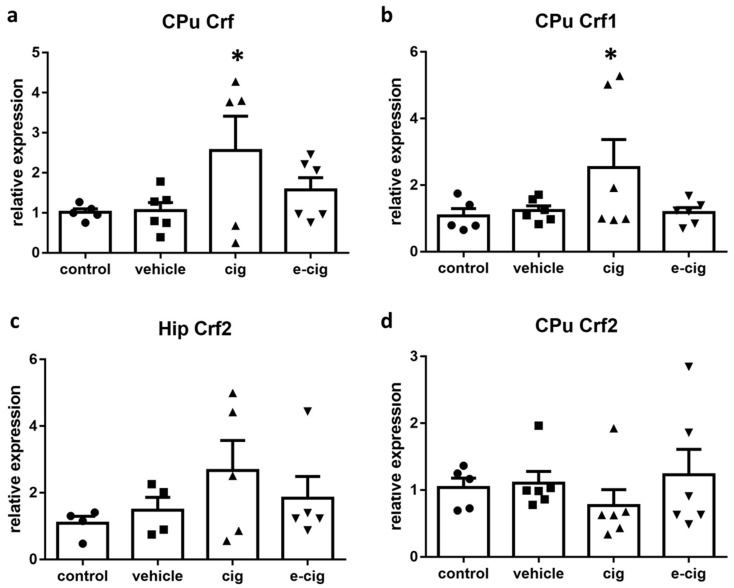
Relative mRNA levels of Crf (**a**), Crf1 (**b**), or Crf2 (**c**,**d**) after e-cigarette (e-cig, downward triangle) or cigarette (cig, upward triangle) exposure with respect to the exposure to air (control, circle) or the e-cigarette administration vehicle (vehicle, square). The results displayed in panels (**a**, **b**, **d**) were obtained in the CPu, while panel c shows the results obtained in the hippocampus. The mice were exposed to cigarettes or e-cigarettes for 7 weeks, followed by a 60-day withdrawal. *n* = 4–6 for each group. *: *p <* 0.05 in the Planned Comparison vs. control. Hip: hippocampus, CPu: caudate-putamen.

Crf and Crf receptors 1 (Crf1) and 2 (Crf2) are regulators of stress responses that elicit anxiety-like behaviours, compulsive drug self-administration, and the stress-induced reinstatement of drug seeking in abstinent animals [14]. The results gathered in our previous studies showed that withdrawal from both tobacco smoke and e-cigarette vapours affected the mRNA levels of Crf and Crf1 in the hippocampus [28]. Therefore, we intended to expand these findings by collecting further data about the Crf system. In contrast to the previous results obtained in the hippocampus, where the 60-day withdrawal from both cigarette smoke and e-cigarette vapours induced a massive decrement of Crf and Crf1 mRNA levels [28], in the CPu a significant increase of Crf and Crf1 mRNA was observed specifically after cigarette exposure (Figure 2a,b). In addition, the mRNA levels for Crf2 were analysed in both brain regions, and no statistically significant alterations were detected (Figure 2c,d). No alterations were observed in the vehicle-treated groups in the hippocampus (not shown, Figure 2c) or CPu (Figure 2a,b,d).

Several studies have demonstrated that Crf mRNA expression is increased in nicotine dependence in the ventral tegmental area, the frontal cortex, and the dorsal striatum [15,29,30,31]. Crf-Crf1 signalling has been repeatedly associated to stressful and anxiety-like behaviours during withdrawal [29,32,33,34], although conflicting evidence is also available [35]. Our previous studies showed that chronic exposure to smoke or e-cigarettes induced an increase in hippocampal Crf and Crf1, which slowly subsided during withdrawal to reach very low levels after 60 days [28]. We interpreted the down-regulation of the hippocampal Crf system accompanied by reduced AMPA receptors as an indication of a loss of neuroplasticity in the hippocampal circuitry, which may support the cognitive and affective impairments detected in the model [28]. The present findings provide the additional piece of information that Crf2 role does not appear to be critical for this effect. Nevertheless, the high variability of the Crfr2 levels within the cigarette group suggests that a differential response could possibly be observed in animal sub-groups. The split distribution could become more evident with a much higher number of subjects than those that were available here; as such, the possibility of an involvement of Crf2 cannot be ruled out in the present condition. However, a distinct role of Crf receptors is in agreement with previous studies, which demonstrated that Crf1 is more implicated in provoking anxiety, dysphoria, and striatal dopamine release [29,32,34,36], while Crf2 demonstrated anxiolytic and antidepressant effects in nicotine withdrawal [37].

In CPu, Crf signalling is reported to strongly increase the spontaneous firing of striatal cholinergic interneurons, thus potentiating dopamine transmission via the activation of acetylcholine receptors on the axons of dopaminergic neurons [38]. In consequence, Crf activation influences the behavioural responses supported by cholinergic transmission in the striatal region. There is accumulating evidence that the dorsal striatum supports cognitive flexibility, namely the shifting of response patterns when environmental conditions change. In the neural circuit that coordinates strategy switching when situations evolve, the dorsal stratum is involved in reliably executing new response patterns, and in inhibiting previous behaviours that are inadequate for the new conditions [39,40]. Indeed, recent findings suggest that the striatal cholinergic interneurons affected by Crf signalling play a critical role in the switching of habitual responses [41].

In drug addiction development, a crucial component is the formation of drug-directed habits that replace goal-directed behaviours. In the early stages, drug seeking is driven by motivated processes, but when the individual becomes addicted, drug seeking behaviours are directly triggered by drug-associated conditioned stimuli, regardless of conscious decisions [42,43]. The voluntary control of drug intake and habit-like drug seeking depend upon different neural networks, with the stimulus-response control over behaviour depending mainly on the dorsal striatum [42]. Therefore, we can postulate that the increased mRNA levels of Crf and Crf1 observed in the CPu after withdrawal from cigarette smoke could be part of a neuroadaptation aimed at modifying drug-related behavioural habits.

### 2.3. Dynorphin System

**Figure 3:**
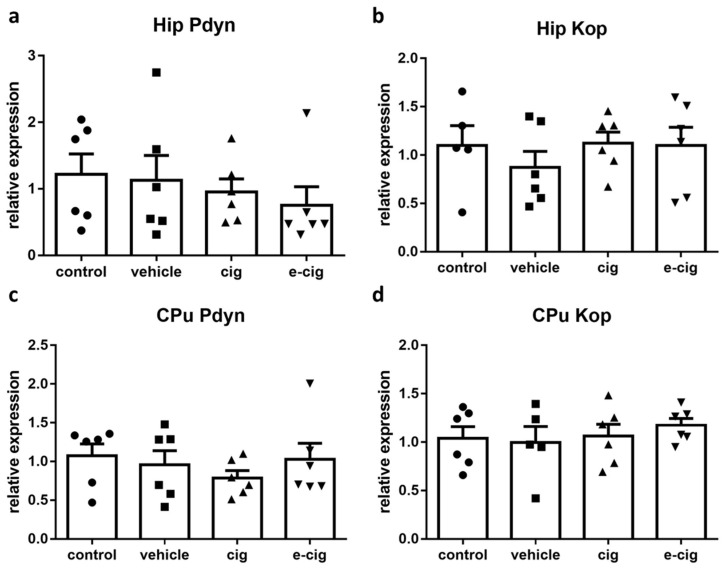
Relative mRNA levels of Pdyn (**a**,**c**) or dynorphin receptor Kop (**b**,**d**) after e-cigarette (e-cig, downward triangle) or cigarette (cig, upward triangle) exposure with respect to exposure to air (control, circle) or the e-cigarette administration vehicle (vehicle, square). The results displayed in panels a and b were obtained in the hippocampus, while panels c and d show the results of the CPu. The mice were exposed to cigarettes or e-cigarettes for 7 weeks, followed by a 60-day withdrawal. *n* = 4–6 for each group. Hip: hippocampus; CPu: caudate-putamen.

Dynophin and its cognate kappa opioid receptor (Kop) are also implicated in the stress response, specifically in establishing the aversive, depressive-like, and dysphoric effects of stress [44]. The available evidence supports a role for dynorphin in the negative affect characteristic of withdrawal, and Kop antagonists have been investigated as potential aids for attenuating its expression [45]. Therefore, we next examined whether the dynorphin-ergic system was altered after 60 days of withdrawal from cigarette smoke or e-cigarette vapours. To this aim, we analysed the mRNA levels of the dynorphin precursor prodynorphin (pdyn) and the Kop receptor. As displayed in Figure 3, the pdyn levels were not altered by withdrawal in the hippocampus (Figure 3a) or in the CPu (Figure 3c). Likewise, the Kop mRNA levels were not modified in the tested regions (Figure 3b,c).

Previous data have shown that nicotine administration increases pdyn levels [13,46,47], but conflicting reports are also available [48,49]. At shorter withdrawal time-points, striatal pdyn levels were reported to be increased [50], while Kop levels were not modified [51], which is in agreement with our results; however, no previous study has investigated 60-day effects. The present findings that, at 60 days of withdrawal, pdyn and Kop were unaltered with respect to the control levels suggests that, at such long-term endpoint, the dynorphin-ergic system is returned at its baseline values, and that its activation is not primarily involved in mediating the affective and cognitive impairments observed. However, whether the dynorphin-ergic system may contribute to withdrawal-related responses by acting on brain regions that were not evaluated in this study cannot be ruled out, as previous data suggest [52].

### 2.4. Nociceptin System

**Figure 4:**
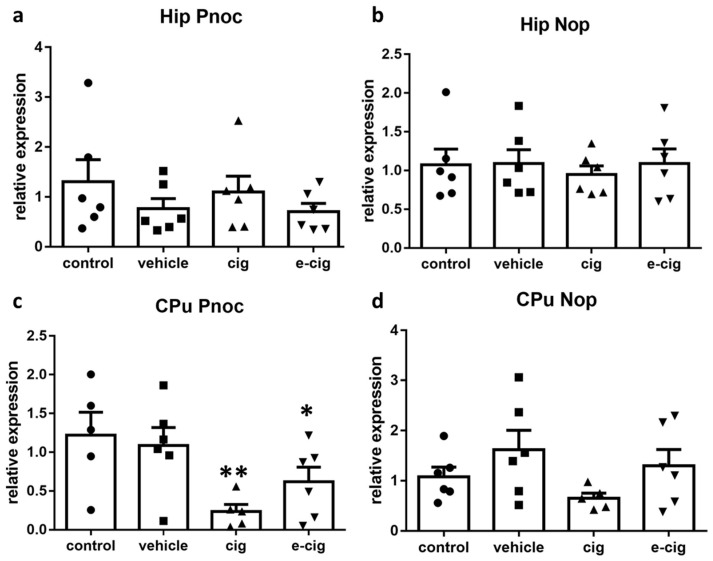
Relative mRNA levels of Pnoc (**a**,**c**) or nociceptin receptor Nop (**b**,**d**) after e-cigarette (e-cig, downward triangle) or cigarette (cig, upward triangle) exposure with respect to exposure to air (control, circle) or an e-cigarette administration vehicle (vehicle, square). The results displayed in panels a and b were obtained in the hippocampus, while panels c and d show the results from the CPu. The mice were exposed to cigarettes or e-cigarettes for 7 weeks, followed by a 60-day withdrawal. *n* = 4–6 for each group. **: *p <* 0.01, *: *p <* 0.05 in the Planned Comparison vs. control. Hip: hippocampus; CPu: caudate-putamen.

Nociceptin (also named Orphanin FQ) and its cognate nociceptive opioid peptide receptor (Nop), which share high similarity to dynorphin and Kop, have been implicated in addiction and withdrawal [53]. Nevertheless, their specific functions have not been adequately elucidated. Indeed, both Nop agonism and antagonism appear to be able to inhibit behaviours related to drug abuse, although antagonism seems to prevail for nicotine [53,54]. Thus, we examined the effects of withdrawal on the nociceptin-ergic pathway by measuring the mRNA levels of both the nociceptin precursor prepronociceptin (Pnoc) and of the Nop receptor. In the hippocampus, no significant difference was induced by nicotine withdrawal in any treatment group (Figure 3a). In contrast, in the CPu, the 60-day withdrawal from both smoke and e-cigarette vapours reduced the Pnoc mRNA levels by 80% and 50%, respectively (Figure 4c). The nociceptin receptor Nop levels were not affected by nicotine withdrawal in any brain region (Figure 4b,d).

Our previous studies demonstrated that Pnoc was increased by repeated nicotine administration in the pre-frontal cortex and the ventral tegmental area, while no alteration was detected in the hippocampus or the striatum [13]. No modifications were observed in Nop levels [13]. The Pnoc and Nop levels in nicotine withdrawal conditions have not been investigated yet, and very little is known about their potential role in this condition. The finding that Nop knockout mice show hypersensitivity to nicotine effects, including withdrawal, supports the hypothesis that this neurotransmission is involved in nicotine addiction [55]. Moreover, Nop agonist treatment elicited an increase of operant behaviour to self-administer nicotine in both dependent and non-dependent rats, while the opposite effect was observed with a Nop antagonist [54]. We can speculate that the reduced Pnoc levels at 60 days of withdrawal are associated with the altered reward learning exerted by the modulation of striatal dopaminergic signalling [56,57].

### 2.5. Enkephalin System

**Figure 5:**
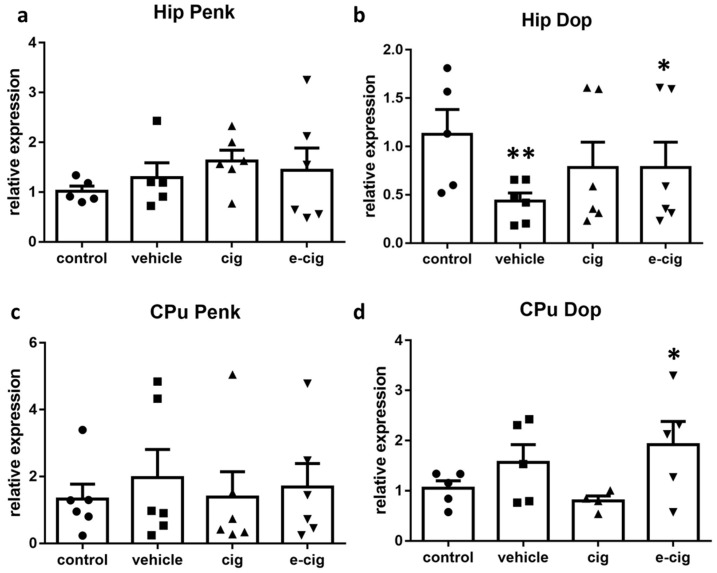
Relative mRNA levels of Penk (**a**,**c**) or enkephalin receptor Dop (**b**,**d**) after e-cigarette (e-cig, downward triangle) or cigarette (cig, upward triangle) exposure with respect to exposure to air (control, circle) or an e-cigarette administration vehicle (vehicle, square). The results displayed in panels a and b were obtained in the hippocampus, while panels c and d show the results of the CPu. The mice were exposed to cigarettes or e-cigarettes for 7 weeks, followed by a 60-day withdrawal. *n* = 4–6 for each group. **: *p <* 0.01, *: *p <* 0.05 in the Planned Comparison vs. control. Hip: hippocampus; CPu: caudate-putamen.

As a next step, we investigated the role exerted by another opioidergic pathway by analysing the mRNA levels of preproenkephalin (Penk), the precursor of enkephalin peptides, and of the enkephalin delta opioid receptor (Dop). The activation of the enkephalinergic system pertains to nicotine-rewarding properties; chronic administration is reported to downplay this pathway as part of neurobiological adaptations to excessive activation, and thus contributes to withdrawal symptoms when nicotine is removed [58,59]. Our results show that Penk mRNA levels were not affected by nicotine withdrawal either in hippocampus or CPu (Figure 5a,c). Surprisingly, the corresponding opioid receptor Dop showed a significant reduction in the hippocampus, which appeared to be driven by the exposure to the e-cigarette administration vehicle (Figure 5b). In contrast, the Dop levels doubled in the CPu of mice that had been exposed to e-cigarettes, with no effect exerted by exposure to cigarette smoke (Figure 5d).

Although they are generally perceived as safe in comparison to cigarettes, e-cigarette vapours can contain harmful substances. In particular, the aerosol from e-cigarettes consists of volatile organic compounds that are derived from solvents and flavours. Growing evidence is becoming available that e-cigarette users are exposed to several toxic compounds which are reported to impact on different organs [60,61]. In particular, the vaporization of propylene glycol and glycerin form the compound acrolein, which is a skin, eye, and nasal irritant, a potential carcinogen, and responsible of the formation of reactive oxygen species (ROS), which have been found in e-cigarette vapours [62]. Since Dop receptors have demonstrated protective efficacy in response to ROS production [63,64], a possible interpretation of the reduced levels observed in the present study is that the modulation represented a response to the ROS formation as a consequence of the long-term inhalation of an e-cigarette vehicle. If it is replicated, this finding will cast further doubts about the alleged safety of e-cigarettes.

### 2.6. Orexin/Hypocretin System

**Figure 6:**
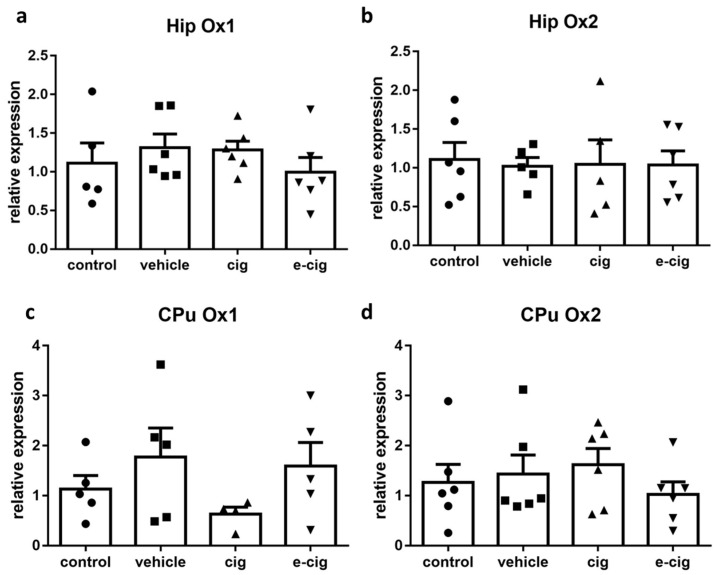
Relative mRNA levels of orexin receptors Ox1 (**a**,**c**), or Ox2 (**b**,**d**) after e-cigarette (e-cig, downward triangle) or cigarette (cig, upward triangle) exposure, with respect to exposure to air (control, circle) or an e-cigarette administration vehicle (vehicle, square). The results displayed in panels (**a**,**b**) were obtained in the hippocampus, while panels (**c**,**d**) show the results of the CPu. The mice were exposed to cigarettes or e-cigarettes for 7 weeks, followed by a 60-day withdrawal. *n* = 4–6 for each group. Hip: hippocampus; CPu: caudate-putamen.

The orexin/hypocretin system consists of the peptides orexin A/hypocretin-1 and orexin B/hypocretin-2, binding to orexin receptors 1 (Ox1) and 2 (Ox2), which have been proposed to coordinate motivational activation in different conditions, including sleep, reward, and stress responses [65]. Accumulating evidence suggests that this system regulates nicotine reinforcement and modulates nicotine’s attention-enhancing properties; moreover, a specific role in withdrawal has been demonstrated for Ox1 [18]. The expression of the orexin precursor was not measured, as it is restricted to hypothalamic nuclei. Our findings showed that no alterations were induced by withdrawal in any orexinergic receptor in the hippocampus or the CPu (Figure 6). These results suggest that Ox1 and 2 are not involved in the withdrawal alterations mediated by the hippocampal and striatal circuits.

### 2.7. Bdnf

**Figure 7:**
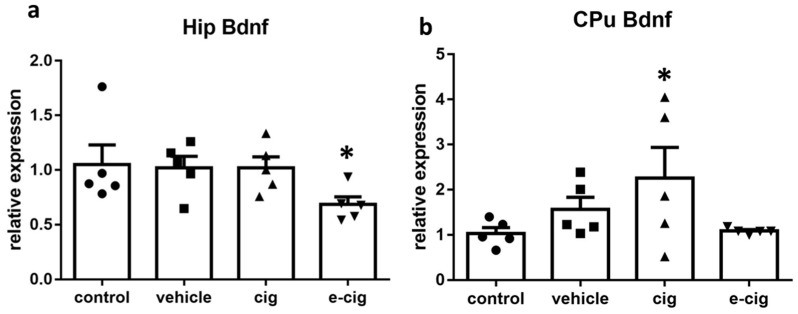
Relative mRNA levels of Bdnf after e-cigarette 8e-cig, downward triangle) or cigarette (cig, upward traingle) exposure with respect to exposure to air (control, circle) or an e-cigarette administration vehicle (vehicle, square). The results displayed in panel (**a**) were obtained in the hippocampus, while panel (**b**) show the results of the CPu. The mice were exposed to cigarettes or e-cigarettes for 7 weeks followed by a 60-day withdrawal. *n* = 4–6 for each group. *: *p <* 0.05 in the Planned Comparison vs. control. Hip: hippocampus; CPu: caudate-putamen.

Bdnf is the member of the neurotrophin family that is the most expressed in the brain. Bdnf regulates proliferation, migration, differentiation and survival during development, whereas, in the adult brain, it activates the pathways regulating synaptic plasticity, synapse formation, and neuronal survival [66]. Therefore, we finally examined the effect of nicotine withdrawal on Bdnf levels, since neurotrophins are involved in the processes underlying the neuroadaptations that mediate the learning and memory of drug-induced behaviours [67]. In the hippocampus, nicotine withdrawal after exposure to e-cigarette vapour induced a 35% decrease of Bdnf levels (Figure 7a). In contrast, Bdnf mRNA was augmented by 118% by cigarette smoke withdrawal in the CPu (Figure 7b).

In the hippocampus, Bdnf levels are reduced in animal models of depression based on chronic stress exposure, and post-mortem studies in depressed patients demonstrated that Bdnf levels are decreased [66,68]. In contrast, treatment with antidepressants is reported to augment Bdnf expression in the hippocampus and pre-frontal cortex, and the up-regulation parallels the time-course of the therapeutic response [66]. Therefore, we suggest that the observed reduction of the Bdnf levels in the hippocampus after e-cigarette withdrawal is implicated in the development of the depressive-like behaviours observed in the model. However, the neuronal plasticity underlying the depressive-like behaviours can be associated to increased Bdnf expression in other brain regions, including the mesolimbic dopaminergic pathway [69]. Within this framework, we can speculate that the increased Bdnf levels in the striatal regions are likewise involved in the depressive-like response observed during withdrawal.

## 3. Materials and Methods

### 3.1. Animals

BALB/cJ mice were obtained from Charles River, Calco, Como, Italy. The animals were housed at five per cage in a humidity- and temperature-controlled animal facility with 12-h light cycle, with lights on at 8.00 a.m. Food and water were freely available. The experimental group assignment was randomly established. Only male mice were used. The experimental procedures respected the guidelines established by the Italian Council on Animal Care, and were approved by Italian Government Decree No. 947/2017-PR. Every effort was made to minimise the number of mice used, and their suffering. The behavioural experiments followed the ARRIVE guidelines [70]. A total of 240 mice were used for the behavioural experiments and the mRNA measures. The animals were submitted to only one behavioural test.

### 3.2. Exposure to Cigarette Smoke and E-Cigarette Vapour

The experimental design is displayed in Supplementary Figure S1. The mice were habituated to the new environment for one week after their arrival, and were randomly split into four experimental groups. The mice were subsequently transferred to plexiglass inhalation chambers (22 cm × 40 cm × 20 cm) connected to a mechanical ventilator (Rodent Ventilator, Model 7025, Ugo Basile, Biological Research Instruments, Varese, Italy), as was previously reported [27,28]. Puffs of vapour, smoke, air, or vapour without nicotine were delivered for three 30-min sessions/day (at 8.00 am, 1.00 pm, 6.00 pm) for seven weeks. The frequency was 25 puffs/min, the flow rate was 200 mL/min, and the volume of each puff was 8 mL. The mice belonging to the cigarette group were exposed to the smoke of 7 commercial cigarettes containing 0.8 mg of nicotine/cigarette (for a total of 16.8 mg/day), 10 mg of tar, and 10 mg of carbon monoxide in each session. The mice belonging to the e-cigarette group were exposed to vapours containing 5.6 mg of nicotine/session (for a total of 16.8 mg/day) dissolved in an aqueous solution containing propylene glycol (55%), glycerin (35%) flavour and fragrance agents (4,7,9-Megastigmatrien-3-one (0.1 mg/mL), 3-ethyl-2-hydroxycyclopent-2-en-1-one (0.7 mg/mL)), Ethyl Maltol (0.67 mg/mL), vanilline (0.15 mg/mL), and neryl acetate (<0.1 mg/mL) [27,28]. The mice belonging to the Vehicle group were exposed to e-cigarette vapours without nicotine. The mice belonging to the control group were exposed to air in three 30-min sessions/day for seven weeks. The investigations were carried out sixty days after the last session of exposure to smoke, vapours or air (Supplementary Figure S1).

### 3.3. Behavioural Studies

All of the behavioural experiments were performed as previously reported [27,28,71]. The tests were performed by three experimenters who were blinded to the treatment. Briefly, in the spatial object recognition test, two visual cues were placed on two adjacent walls of an opaque white plexiglass cage that was dimly lit from above the cage. Two groups of identical objects were used. The objects in one group were inverted 50 mL falcon tubes filled with clean mouse bedding; the other group’s objects consisted of yellow and green plastic interlocking blocks built into 10 cm towers. The object’s types and positions were randomly switched. The day before the experiment, immediately after the last exposure to smoke or vapours, the mice were pre-exposed to the cage for 10 min. After one day, the mice were returned to the cage and the time spent exploring the two objects was recorded by a camera mounted above the cage. Thirty seconds were allowed for exploration. Exploration was defined as a mouse having its nose directed toward the object within approximately 1 cm. Climbing or sitting on objects was not considered to be exploration. After forty-eight hours, the mice were placed in the cage in which the more explored object had been moved to a different position.

In the tail-suspension test, the mice were habituated to the test room for one hour before the experiment started. A paper adhesive tape was placed 1 cm from the tip of the tail, and the mice were individually suspended 35 cm above a table top. The behaviour of the mice was video camera-recorded for six minutes, and the immobility duration was measured by a trained observer blinded to the treatment groups. The mice were considered immobile when they hung passively and completely motionless. The mice that climbed their tails were excluded from the data analysis.

In the sucrose preference test, the mice were individually housed for three days in a free choice condition between two bottles containing either tap water or 3% sucrose in tap water. The bottle position was switched every 24 h. The intake was assessed by weighing the bottles every day, and the sucrose preference was calculated as a percentage of the sucrose solution to the total liquid intake through the three days.

Anxiety was evaluated using the marble burying test, which utilizes spontaneous digging behaviour—which is characteristic of rodents—to assess anxiety-like/compulsive behaviour. After acclimation (1h), each mouse was placed in a cage in which 20 marbles had been equally distributed on top of mouse bedding (5 cm in depth). After 15 min, the number of buried marbles and the latency to the first marble burying were counted.

### 3.4. mRNA Level Studies

After 7 weeks of smoke or vapour exposure, 6 mice per group were selected for the mRNA level studies. The mice were euthanized by means of cervical dislocation. Their brains were rapidly removed and placed on ice so that the brain regions could be dissected out. Firstly, the hippocampus was manually isolated and dissected out, and then a slice from the rostral end of striatum to the optic chiasm was used to dissect the CPu. The brain regions were quickly frozen on dry ice before being stored at −80 °C.

Real-time quantitative PCR experiments were carried out following the previously published methods [15,28], with some modifications. The Aurum total RNA fatty and fibrous tissue kit (Bio-Rad, Hercules, CA, USA) was used for the RNA extraction from the hippocampus and CPu. The RNA quantification was performed by ultraviolet (UV) absorbance in a NanoDrop 2000c spectrophotometer (ThermoFisher Scientific, Waltham, MA, USA), followed by agarose gel electrophoresis to confirm the RNA’s integrity. The cDNA synthesis was achieved using the iScript Advanced cDNA synthesis Kit (Bio-Rad). The real-time PCR was performed by Sybr Green technology in a 7900HT Fast Real-Time PCR System (Applied Biosystems, Thermofisher Scientific) with Sso Advanced Universal SYBR Green Supermix (Bio-Rad) according to this protocol: stage 1: 95 °C, 20 s; stage 2: 40 × (95 °C, 3 s; 60 °C, 30 s). The primer sequences were the following: Crf Forward 5′-GGAGCCGCCCATCTCTCT-3′; Crf Reverse 5′-TCCTGTTGCTGTGAGCTTGCT-3′; Crf1 Forward 5′-GATCAGCAGTGTGAGAGCCT-3′; Crf1 Reverse 5′-GTTGTAGCGGACACCGTAG-3′; Crf2 Forward 5′-GGGCTTTACCTTGGTGGGTAG-3′; Crf2 Reverse 5′-TGCTTCCAAAGGAGGTCTGTC-3′; Pdyn Forward 5′-GGCTACAGTGCACTAGCCAA-3′; Pdyn Reverse 5′-GCTGGTAAGGAGTCGGCTTT-3′; Kop Forward 5′-GTTCCCCAACTGGGCAGAAT-3′; Kop Reverse 5′-AATTGCCCACTAAGCCCACC-3′; Pnoc Forward 5′-GGCAGGATTGTAGTTGGGCT-3′; Pnoc Reverse 5′-TTCCCTGCCTACCTCGATGA-3′; Nop Forward 5′-GGGCCCTGTATTTGCCATCT-3′; Nop Reverse 5′-CTACCAGTACCAGCCGTGTG-3′; Penk Forward 5′-GCAGGAAACACTAGGGTCCAA-3′; Penk Reverse 5′-GTAGGAGAGAAGAACGCGCC-3′; Dop Forward 5′-CACGGTGGAGACGGACAC-3′; Dop Reverse 5′-AAAGGCGTCCGAGAGGTTG-3′; Ox1 Forward 5′-CACTACCGTGCTGTCCTGAG-3′; Ox1 Reverse 5′-GCTTAAGCCCCTATCCACCC—3′; Ox2 Forward 5′-GACCACACAGATCAGCAACTT-3′; Ox2 Reverse 5′-AGATTCCATAAGGATGCTCGGG-3′; Bdnf Forward 5′-GAGGCTAAGTGGAGCTGACAT-3′; Bdnf Reverse 5′-TTCCCCACCTCCATCCTAGA-3′; Gapdh Forwad 5′-GCCAAGGTCATCCATGACAACT-3′; Gapdh Reverse 5′-GAGGGGCCATCCACAGTCT-3′; Ywhaz Forward 5’-TAGGTCATCGTGGAGGGTCG -3’; Ywhaz Reverse 5’-GAAGCATTGGGGATCAAGAACTT -3’. The primers were obtained from Eurofins, Vimodrone, Italy. The data were analysed using the Delta-Delta-Ct method, and were converted to a relative ratio (2-DDCt) for the statistical analysis [72] by normalizing to the geometric average of the endogenous reference genes Gapdh and Ywhaz. The specificity of the amplification product was evaluated by building a dissociation curve in the 60–95 °C range.

### 3.5. Statistical Analysis

The data are presented as the observed mean values ± SEM. The spatial object recognition data were analysed using a 2-way repeated measures mixed model approach, with the Treatment (Control, vehicle, cig, and e-cig) as the treatment factor and the Time-point (24 h or 48 h) as the repeated factor. The other responses were analysed using a 1-way ANOVA approach with the Treatment (Control, vehicle, cig, and e-cig) as the treatment factor. As the samples were analysed in different plates using a complete block design, an additional blocking factor Plate was also included in the statistical model in order to account for any plate-to plate variability [73]. The analyses were followed by Planned Comparisons of the predicted means in order to compare the mean of the cigarette, e-cigarette and vehicle groups to the mean of the control group. The analysis was performed using the InVivoStat v4.1.0 software [74]. The data were log- or square-root-transformed, where appropriate, in order to stabilize the variance and satisfy the parametric assumptions. A value of *p <* 0.05 was considered to be statistically significant.

## 4. Conclusions

This study shows that the long-term withdrawal symptoms belonging to both the affective and cognitive domains can be reproduced in mouse models of both cigarette smoking and e-cigarette vaping. A number of limitations should be kept in mind. Since the investigation was designed with the objective of deriving new hypotheses about the molecular correlates of behavioural impairments to be further tested in future experiments, a relatively small number of biological replicates were considered and no correction for multiple comparisons was drawn, thus increasing the chance of type-1 errors. Likewise, only male animals were used; as such, the conclusions cannot be extended to females. Furthermore, whether the mRNA level alterations were translated into different protein amounts was not assessed. Moreover, during the sucrose preference test procedure, the mice were singly housed, and the exposure to this additional stressor may have influenced the test results. As strong points, the experimental design of these models is aimed at mimicking, as closely as possible, the intermittent smoke or vapour exposure maintained for a long duration as it happens in humans. Indeed, the affective and cognitive impairments detected in the present study reflect the long-term consequences of nicotine withdrawal as it is observed in human abstinent smokers. The observed long-term behavioural alterations are associated with lasting molecular alterations in peptidergic signalling. In particular, Crf, Crf1, and Bdnf reduction in the hippocampal region are suggested to determine a hampered neuroplasticity in the hippocampal circuitry, which may be responsible for the cognitive and affective impairments detected in the model. In addition, the alterations in Crf, nociceptin, enkephalinergic, and Bdnf transmission in the CPu may be involved in the maladaptive habit learning associated with nicotine-seeking behaviours, which are maintained for a long period of time. Overall, these data show that e-cigarette use is able to provoke long-term alterations in brain signalling that are comparable to those of cigarette smoke, indicating that the investigation of neurobiological correlates of e-cigarette vaping deserves further research efforts. It must indeed be underlined that several differences between cigarettes and e-cigarettes emerged between the long-term effects of withdrawal on striatal and hippocampal neurochemistry, and that—in some instances—the e-cigarette vehicle had significant effects by itself. This evidence suggests that other compounds besides nicotine contribute to the long-term effects of chronic cigarette and e-cigarette exposure.

## Data Availability

The data presented in this study are available in the article.

## Supplementary Materials

The following are available online at https://www.mdpi.com/1422-0067/22/2/599/s1.

## Abbreviations

cig	Cigarette
CPu	Caudate-putamen
Crf	Corticotropin-releasing factor
Crf1	Corticotropin-releasing factor receptor 1
Crf2	Corticotropin-releasing factor receptor 2
Dop	Delta opioid receptor
e-cig	Electronic cigarette
Kop	Kappa opioid receptor
Nop	Nociceptive opioid peptide
Ox1	Orexin receptor 1
Ox2	Orexin receptor 2
Pdyn	Prodynorphin
Penk	Preproenkephalin
Pnoc	Prepronociceptin
ROS	Reactive oxygen species
