# Supplementary material for: Altered mRNA Levels of Stress-Related Peptides in Mouse Hippocampus and Caudate-Putamen in Withdrawal after Long-Term Intermittent Exposure to Tobacco Smoke or Electronic Cigarette Vapour
Source: Int J Mol Sci. 2021 Jan 9;22(2):599. doi: 10.3390/ijms22020599 (PMC7827390; doi:10.3390/ijms22020599)
Supplement: Supplementary file 1 [file ijms-22-00599-s001.pdf]

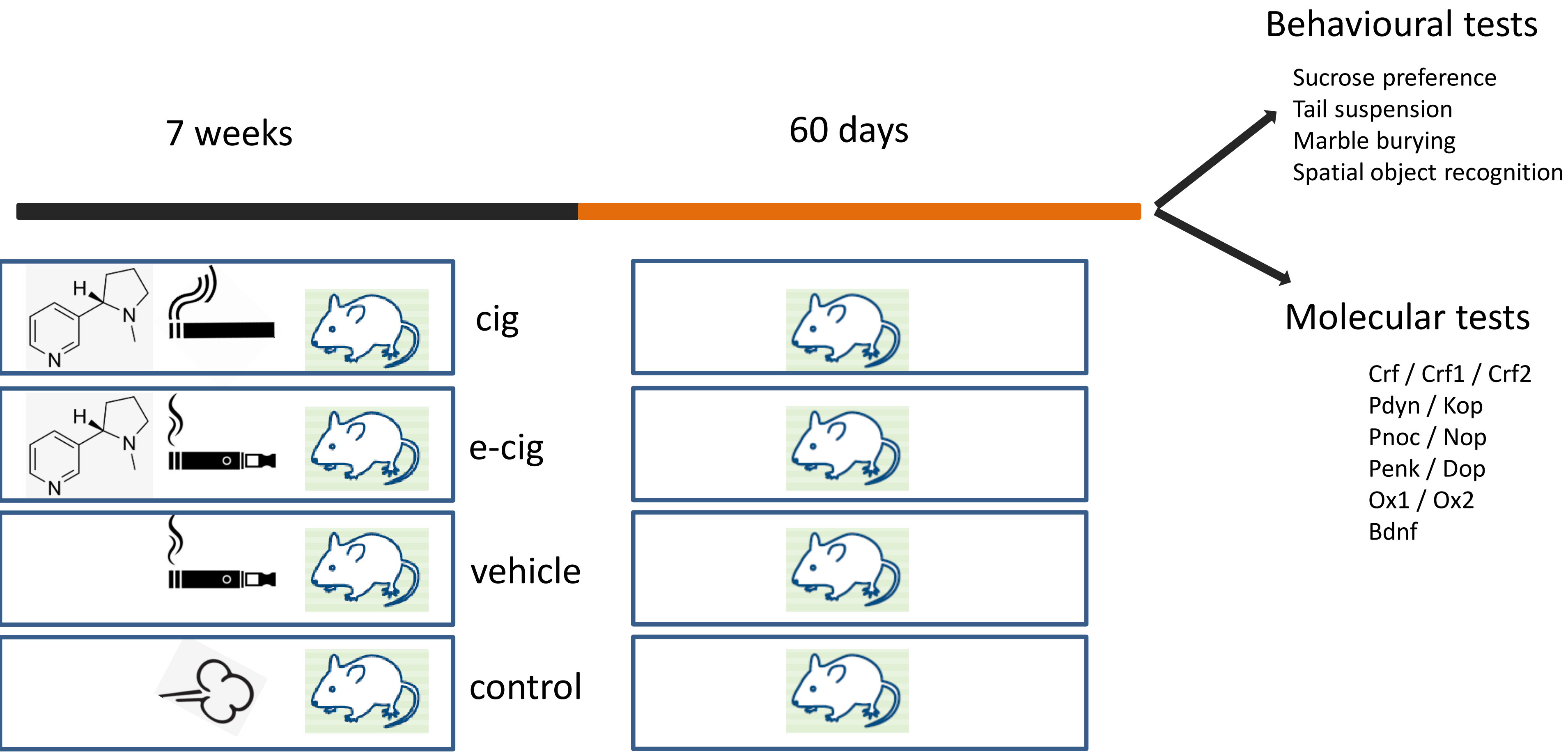
Behavioural tests
Sucrose preference
Tail suspension
Marble burying
Spatial object recognition
7 weeks
60 days
H
N
N
cig
e-cig
vehicle
control
Molecular tests
Crf / Crf1 / Crf2
Pdyn / Kop
Pnoc / Nop
Penk / Dop
Ox1 / Ox2
Bdnf
